# Virtual reality exergame for supplementing multimodal pain therapy in older adults with chronic back pain: a randomized controlled pilot study

**DOI:** 10.1007/s10055-022-00629-3

**Published:** 2022-02-11

**Authors:** Oskar Stamm, Rebecca Dahms, Norbert Reithinger, Aaron Ruß, Ursula Müller-Werdan

**Affiliations:** 1 Department of Geriatrics and Medical Gerontology, Working Group Age and Technology, Charité - Universitätsmedizin Berlin, Corporate Member of Freie Universität Berlin and Humboldt-Universität zu Berlin, Reinickendorfer Straße 61, 13347 Berlin, Germany; 2 DFKI, Deutsches Forschungszentrum Für Künstliche Intelligenz GmbH (DFKI), Alt-Moabit 91c, 10559 Berlin, Germany

**Keywords:** Physical therapy, Psychotherapy, Virtual reality, Multimodal pain therapy, Serious gaming, Chronic back pain

## Abstract

Immersive Virtual Reality (VR) with head-mounted displays (HMD) can be a promising tool for increasing adherence to exercise in older adults. However, there is little known about the effectiveness of an interactive multimodal therapy in VR for older chronic back pain (CBP) patients. The aim of the exploratory randomized controlled trial was to examine the preliminary effectiveness of a VR multimodal therapy for older adults with CBP in a laboratory setting over a period of four weeks. The intervention group (IG; *n* = 11) received a multimodal pain therapy in VR (movement therapy and psychoeducation) and the control group (CG; *n* = 11) received a conventional multimodal pain therapy (chair-based group exercises and psychoeducation in a group setting). Although the VR therapy (IG) did not reach the pain intensity reduction of the CG (IG: MD = 0.64, *p* = .535; CG: MD = 1.64, *p* = .07), both groups showed a reduction in pain intensity on the Numeric Rating Scale. The functional capacity in the IG improved from Visit 1, $\overline{x }$ = 73.11% to Visit 2, $\overline{x }$ = 81.82% (MD = 8.71%; *p* = .026). In the changes of fear avoidance beliefs and general physical and mental health, no significance was achieved in either group. Although the IG did not reach a significant pain intensity reduction compared to the CG, the results of the present study showed that a pain intensity reduction can be achieved with the current VR application.

## Introduction

Back pain is one of the most common musculoskeletal conditions. Globally, low back pain has been the leading cause of disability measured by years lived with disability (Vos et al. 2017). When the pain persists for more than 12 to 24 weeks, a chronic course can be assumed (Dionne et al. 2008). In Germany, the highest prevalence of chronic back pain (CBP) is found among adults aged 70 years and older (28%) (Von Der Lippe et al. 2021). CBP is a complex multidimensional disorder, in which kinesiophobia, fear-avoidance beliefs and passive coping strategies may often occur (Waddell 2004; O’Sullivan 2012). Therefore, multidisciplinary treatment programs including psychological interventions in addition to physical treatment have become standard in the treatment of CBP patients. Most guidelines recommend behavioral interventions, several recommend considering fear-avoidance beliefs (Reese and Mittag 2013). Systematic reviews found evidence of moderate quality in terms of the effectiveness of behavioral therapy for chronic low back pain (Brox et al. 2008; Baez et al. 2018). Research indicates that patients with CBP receiving multimodal pain therapy and rehabilitation experienced less pain intensity (0.5 to 1.4 units on the Numeric Rating Scale) and disability (1.4 to 2.5 points on the Roland Morris Disability Questionnaire), and a greater increase in their physical function, compared with patients receiving a standard treatment that focuses only on physical function (Pfingsten et al. 1997; Kamper et al. 2014).

CBP programs performed by patients alone could be facilitated by new technologies (Palazzo et al. 2016). So-called exergames (exercise games) might be a potential approach to adherence enhancement and could provide adjuvant therapy to multimodal pain management. Studies indicate that exergames can lead to an increase in motivation and can be a promising tool for increasing adherence to exercise in older adults (Brox et al. 2011; Meekes and Stanmore 2017). Recent studies showed that a specially developed Nintendo Wii exergame could be a biopsychosocial intervention for chronic low back pain (Graves et al. 2010; Park et al. 2013; Kim et al. 2014). Hoffman et al. (2000) have shown in early scientific work with immersive virtual reality that VR can distract from high levels of pain during wound care. Immersive Virtual Reality (VR) with head-mounted displays (HMD) offers new opportunities in exercise therapy. In the systematic review of Mallari et al. (2019), there was some research that suggested VR can reduce chronic pain during the intervention. Jones et al. (2016) showed a significant decrease in pain during a low-motion VR game application on chronic pain. However, there is a dearth of studies using VR active exercise therapy (Villafaina et al. 2019; Cao et al. 2021; Kruse et al. 2021) and psychotherapy (Fodor et al. 2018) together. Most of the VR studies in chronic pain patients are using distraction as a pain reduction technique. Also vision has been used to augment the embodied experience. Visual feedback by watching the site of the chronic back pain may be helpful in alleviating the pain (Diers et al. 2016). An initial therapy study with exercises in VR by Alemanno et al. (2019), using a six-week VR treatment to teach patients to execute correct movements with the painful body parts, showed significant reductions in pain rating scale scores and significant improvements of quality of life in the domains of physical functioning.

However, there are no studies investigating an active VR exergame with an HMD for older CBP patients. In order to secure an effective long-term therapy for CBP patients, a multimodal approach in VR is necessary. Within the scope of the ViRST project, we determined measurements for various physiological outcomes, but also offer psychological exercises and behavioral recommendations. Based on a requirements analysis (Stamm et al. 2020), the ViRST application was developed in a two-year project. The aim of the evaluation was to examine changes in pain intensity, functional capacities and fear-avoidance beliefs. Within this evaluation, the researchers aimed to answer the following research questions:

### Primary research question

Does the VR system contribute to an effective, multimodal pain therapy for the treatment of CBP in older adults? The following aspects will be considered:

- Changes in pain intensity and severity of chronic pain
- Changes in functional capacities
- Changes in fear-avoidance beliefs (kinesiophobia)

### Secondary research questions

What impact does the use of the VR system have on the health-related quality of life of the older adults with CBP patients after usage?

- Changes in general physical and mental health
  How do older people with CBP rate the user experience of a four-week multimodal pain therapy in virtual reality?
- Rating of the degree of the immersion
- Evaluation of the user experience

## Material and methods

### Study design

In the monocentric study, we conducted randomized controlled pilot trial with a parallel arm design (1:1 allocation ratio) in older adults with CBP. The study took place between January and March 2020 under laboratory conditions to compare the impact of a VR exergame on pain intensity progression, functional capacities, fear-avoidance beliefs and general physical and mental health with a standard practice (multimodal pain therapy). In addition, the user experience was evaluated regarding the use of VR in therapy. Important changes to methods after the trial took place due to the COVID-19 pandemic and the associated lockdown and contact restrictions; the maximum force measurement could not be performed with the intervention group in Visit 2 and it does not appear as part of the data analysis. Ethical approval was gained from Ethics Committee of the Charité – Universitätsmedizin Berlin (No. EA4/213/19). The trial is registered at the German Clinical Trials Register (DRKS-ID: DRKS00020576).

### Study population

We applied the following inclusion criteria: (1) CBP for longer than six months, (2) being 65 years of age or older, (3) independent mobility, (4) able to actively perform exercises, (5) no intervertebral disc surgery in medical history and (6) no severe vestibular restrictions affecting the ability to balance. We excluded candidates with (1) immobility or those whose mobility was possible only with assistance, (2) with sensory and motor failure, (3) with spinal malignancies, spondylitis or spondylodiscitis, (4) severe vestibular impairment which effects the ability to balance, such as dizziness or severe visual impairment (oscillopsia).

### 2.3 Procedure

We assessed the eligibility of all volunteers prior to the start of the study via personal telephone screening. Subsequently, the subject information was sent to the candidates by mail or e-mail and all screened subjects had at least 24 h to decide whether or not to participate. If the criteria for inclusion in the study were met, the participants were then sent the subject information. Informed consent was obtained from all participants before enrollment. The study was conducted in the facilities of the research group at the Charité and the Sport-Gesundheitspark Berlin.

Data collection was limited to conducting the survey with older adult patients with CBP at different times: Visit 1: approximately 45 min; intervention phase: four weeks; Visit 2: approximately 45 min. Before the intervention phase started, an anamnesis interview and an orthopedic examination with a sports scientist and physiotherapist took place to identify possible contraindications for testing and further study continuation. This was a one-off examination lasting about 30 min. Subsequently, questionnaires were given to the participants in Visit 1.

During the intervention phase, each group was provided with multimodal pain therapy for CBP patients three times a week for about 30 min. A total of 12 exercise units were offered to each participant on a voluntary basis. This training consisted of both physiotherapeutic and psychotherapeutic exercise units. In order to determine the course of pain, a pain diary was used before and after each exercise session, i.e., ideally a total of 24 times. At the end of the intervention phase, i.e., in the last unit (Visit 2), the same questionnaires were given to the participants again.

### Study interventions

The participants were divided into an intervention group (IG; VR exergame) and a control group (CG; chair-based group exercises). The IG received a multimodal pain therapy in VR (movement therapy and psychoeducation) for four weeks, with three appointments per week lasting approximately 30 min in a laboratory setting. The CG completed a four-week conventional multimodal pain therapy (movement therapy as seated exercises and psychoeducation in a group setting) for four weeks, with three appointments per week lasting approximately 30 min.

### IG (VR exergame)

In the course of the intervention phase, attention was paid to the implementation of a multimodal concept for the treatment of back pain patients, which had a physiotherapeutic and psychotherapeutic focus. A training session of the IG was conducted by participants under physiotherapeutic supervision with a VR HMD headset using the ViRST VR application, which was developed in the course of the ViRST research project. The training session, consisting of 12 exercises, was structured as follows: (1) warm up (training of the upper and lower extremities); (2) main part (strengthening of the abdominal and back muscles, core stability); (3) cool down (stretching, progressive muscle relaxation exercise); (4) psycho-educative units (topics: physiology of pain, pain management, stress management, everyday training). The IG psychoeducative units were always shown using the VR headset at the end of each training week.

### CG (chair-based group exercises)

The intervention phase in the CG differed from IG mainly in the use of VR technology. The participants in the CG took part in conventional sitting gymnastics consisting of 12 exercises identical to those in the VR group, but under the guidance of a physiotherapist in a circle of chairs in small groups rather than individually using VR. Depending on the participants' preference for a timed session, a maximum group size of six participants was targeted. The psychoeducative units (physiology of pain, pain management, stress management, everyday training) were offered at the end of a training week, analog to the IG. The psychoeducation was provided in the CG in a conservative manner using interactive patient training, in which a therapist presented information on a flip chart.

### Outcomes

The primary outcome was a composite outcome, which included change in pain intensity and the severity of chronic pain, changes in functional capacities, changes in fear-avoidance beliefs (kinesiophobia) and changes in the maximum strength of the trunk muscles and muscular imbalances (not further described, unable to complete because of COVID-19). For this purpose, the following validated assessments were applied: the Numeric Rating Scale (NRS) (Hilfiker 2008) to assess current pain intensity; the Chronic Pain Grade Questionnaire (CPGQ) (Von Korff et al. 1992) to assess the severity of chronic pain; Hannover Functional Ability Questionnaire for measuring back pain-related disability (Ffb-H-R) (Kohlmann and Raspe 1996) to assess functional capacities; the Tampa Scale of Kinesiophobia (TSK-11) (Rusu et al. 2014) to assess fear-avoidance beliefs.

As secondary outcomes, we investigated general physical and mental health with the Health Survey SF-12 (Ware et al. 1996), the immersion of the applied VR system with the Technology Usage Inventory (TUI) (Kothgassner et al. 2012) and the user experience with the User Experience Questionnaire (UEQ) (Laugwitz et al. 2008).

### Measures

#### NRS

The participants' pain intensity progression was recorded in a pain diary using the NRS, in which the current pain intensity before and after each training session was recorded. The NRS is a scale to assess self-reported pain intensity that ranges from 0 to 10, where 0 means ‘no pain’ and 10 means ‘the worst pain imaginable.’

#### CPGQ

CBP was assessed by the CPGQ. The questionnaire by von Korff et al. assesses the severity of chronic pain. All items are rated on an 11-point Likert scale. The scores enable the classification of chronic pain into functional chronic pain (grades I and II) and dysfunctional CBP (grades III and IV). Grade 0 means no pain, grade I means low disability-low intensity, grade II means low disability-high intensity, grade III means high disability-moderately limiting and grade IV means high disability-severely limiting. The translated German version of the CPGQ was used.

#### Ffb-H-R

Both groups received the Ffb-H-R for measuring back pain-related disability. This questionnaire by Kohlmann and Raspe serves to assess the functional limitations in activities of daily living due to back pain. It is a self-report instrument consisting of 12 items, each with three response options. The result of the evaluation represents the functional capacity in percent of the patient, where 100% means the maximum and 0% means the minimum of functional capacity. The German version of the Ffb-H-R was used.

#### TSK-11

The TSK is one of the most commonly used measures of pain-related anxiety in back pain patients. We used the shortened version of the TSK, the TSK-11 (Woby et al. 2005). The items of the TSK-11 are scored from 1 (strongly disagree) to 4 (strongly agree), giving a total score of 11–44 points, with higher scores indicating greater pain-related fear.

#### SF-12

The SF-12 is a shortened version of the SF-36. The SF-12 provides two scores: the physical scale score and the mental health score. Possible scores range from 0 to 100 points, with 0 representing the greatest possible health limitations and 100 representing the absence of health limitations. The SF-12v2 in German was used.

#### TUI

The TUI is used to assess technology-specific and psychological factors that contribute to the actual use of a technology. The instrument contains the following eight scales: Curiosity, Anxiety, Interest, Ease of Use, Immersion, Usefulness, Skepticism and Accessibility. In addition, the procedure contains the Intention to Use (ITU) scale. This paper will discuss Immersion in more detail, which was applied in the IG.

#### UEQ

The UEQ measures the user experience of interactive products and the full version in German was used. It examines the valence dimension ‘attractiveness,’ which is subdivided into two quality aspects: pragmatic quality (Perspicuity, Efficiency, Dependability) and hedonic quality (Stimulation, Novelty). The UEQ contains six scales of the quality aspects with 26 items scaled from −3 (most negative) to + 3 (most positive).

### ViRST VR game

**Fig. 1:**
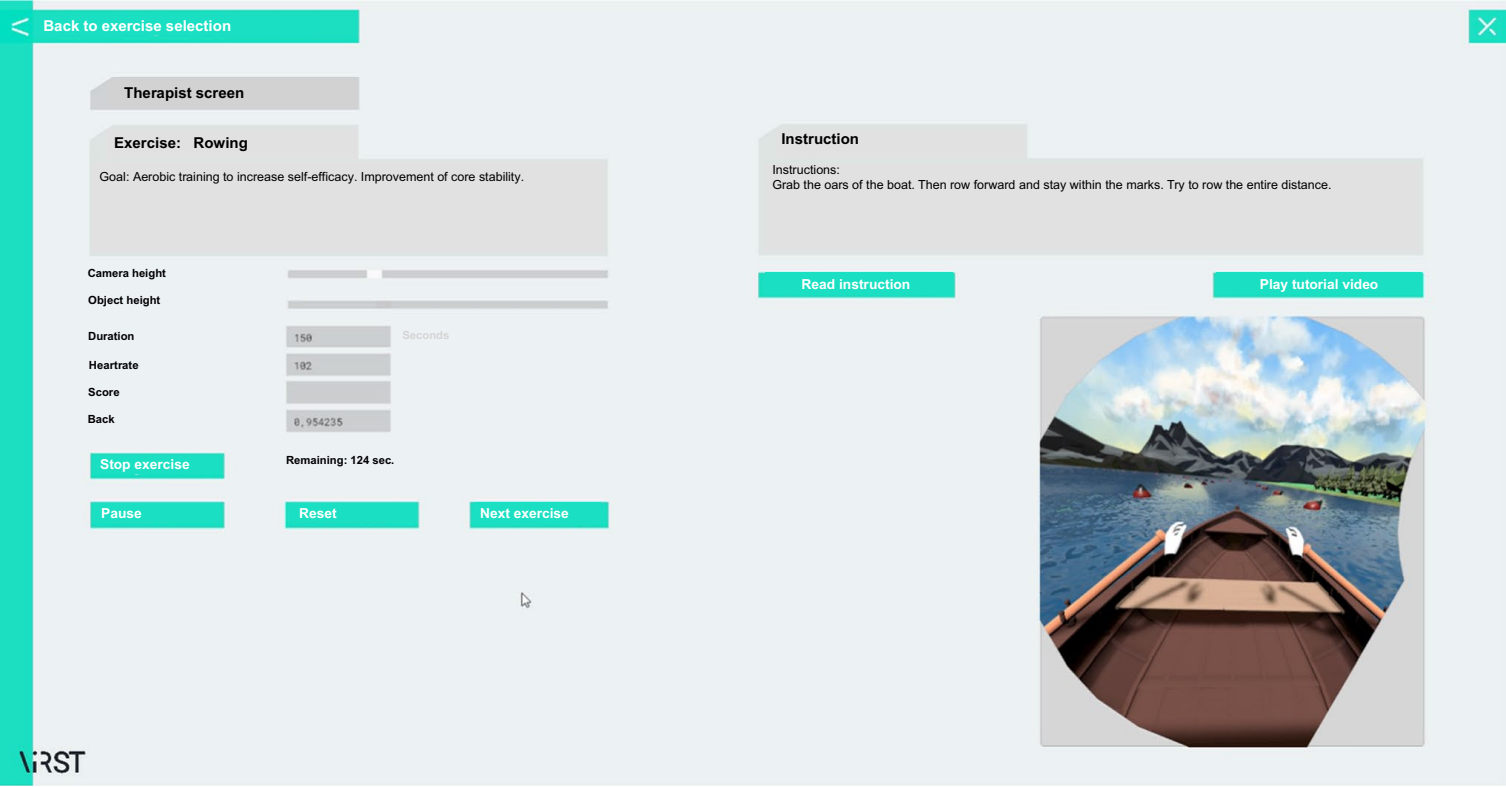
Therapist user interface

**Fig. 2:**
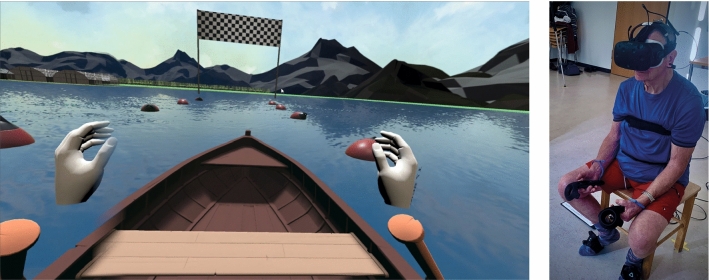
Left: user view of the VR game, right: patient set-up

The developed VR game was composed of two software interfaces: a therapist interface (Fig. 1), which allows for setting the exercises and monitoring the live image of the participant, and the VR game (Fig. 2), in which the participant performs interactive tasks on a farm (e.g., rowing, turning on light bulbs, or sorting vegetables). The desired effect on the user during gameplay was to achieve an ‘immersiveness’ of the environment, i.e., the player is immersed in a computer-generated environment and temporarily perceives it as real. For this purpose, an HTC-Vive VR system (consisting of VR headset and two controllers) and a laptop were used. The interaction between the user and the VR system was facilitated by a speech-based dialog system that guided the user through the exercise. To avoid excessive strain, we integrated a real-time stress assessment by photoplethysmography (PPG). Detected changes in the cardiac rhythm are classified with regard to strain. The heart rate was displayed on the therapist interface in real-time and also served as a control for the therapist. In case of exceeding the target training heart, the dialog system interacts with the user, e.g., to pause the game. The training heart rate was 0.75 max HRR + resting HR; where max HRR is the maximum Heart Rate Reserve, and resting HR is resting Heart Rate (Kent 2007). However, this was not exceeded in any case during the test. The dialog system uses off-the-shelf speech recognition and synthesis modules. The hybrid dialog control module merges automaton based and Neural Network based approaches. The PPG stress sensor was trained on the heart rate signals collected from participants.

The warm-up included the exercises: “marching” and “rowing.” The aim of the marching on the spot was the activation of the circulation of the lower extremities. Here, the users had to step on buttons coming toward them on the floor in a certain rhythm. Trackers attached to the feet allowed users to interact with the virtual world. In the second warm-up exercise, participants rowed on a lake and had to maintain a course between buoys (Fig. 2). To do this, the participants had to grip the oars with the HTC Vive controllers by pressing the trigger button. Once the oars had been gripped, they remained fixed to the virtual hands. This exercise serves to activate the circulation of the upper extremity.

The first exercise in the main part was the “balloon pump.” This exercise was designed to strengthen the back extensors. In this exercise, the participant bent down and came back up halfway. There they performed small forward and backward movements with a straight back. In VR, the participant saw an air pump with which they blew up balloons. The next exercise was called “hurdles.” Here, the participants lifted both feet while sitting and kept abdominal tension and their balance. Through the trackers on the feet, they could see their virtual feet. While lifting the feet, they jumped over hurdles in virtual reality. The goal of the exercise was to strengthen the abdominal muscles. The subsequent exercise: “the bridge” focused on improving core stability and strengthening chest and shoulder muscles. With their bodies bent forward, participants rocked forward on a bridge over the lake by pulling forward on the bridge's ropes through trigger buttons of the controllers. In the “light bulbs” exercise, participants climbed a ladder at the farmhouse by grasping the rungs through the trigger button. Once at the top, they screwed in light bulbs with the controllers, by releasing the triggers the light bulb could be released. The focus was on mobilizing the shoulder joint as well as the cervical and thoracic spine. In the “shaking bottles” exercise, the user's shoulder blades were fixed while the arms were pushed through at the elbow. Then small quick movements were made with the controllers out of the shoulder. The participants shook bottles in the VR until the corks popped. The exercise was designed to strengthen the deep back muscles and improve core stability. In the “ball bucket,” the participant stretched both arms out to the side with the controllers in their hands and tries to make small quick movements up and down with the arms. Abdominal muscle tone was built and one leg was lifted. After a while the other leg was lifted while continuing small arm movements. The participants had the task in VR to empty the self-filling buckets as quickly as possible. The exercise was designed to strengthen the deep back muscles and improve core stability.

The next exercises were part of the cool-down. First, the focus was on stretching the rotational muscles. In “vegetable sorting,” participants stood in front of a conveyor belt and had to take turns sorting vegetables into a box on the right and left. By pressing the trigger, they were able to grab the vegetables. The goal was to stretch the serratus anterior muscle and the pectoralis major muscle. In the boiler exercise, the participants stood next to the farmhouse in the VR and had to regulate the temperature in the boiler. To do this, they had to use the controllers to push one handle up and one handle down. This exercise was designed to stretch the latissimus dorsi muscle and the quadratus lumborum muscle. In the “apple tree” exercise, the arms were brought together above the head in a seated position and stretched upwards. Then, the upper body is tilted to one side at a time. In VR, the participant imitates the movements of a tree. The last exercise was progressive muscle relaxation. The participant learned how the muscles feel when tense by tensing the hands and arms and relaxing the muscles after a while. In VR, the participant stands on a dock by the water, accompanied by relaxation music, and imagines warmth by making a fist, symbolized by coloring his fists red. Tightening the muscles is done by firmly grasping the controllers, while the grip button was pressed. The psychoeducational sessions were shown through the VR headset at the end of each week in the form of interactive videos.

### Randomization

The assignment of the participants to a group was done with a randomized block design. To obtain as comparable a study group as possible, randomization was stratified with respect to gender. A unique number was generated for each participant after eligibility screening by the study investigators. Randomized participants received therapy during the study period according to the intervention they were allocated.

#### Sample size

Moore et al. (2011) recommend at least 12 participants for pilot studies to conduct within single centers to provide valuable preliminary information. With a sample size of 22 participants, we were considerably above the rule of 12.

#### Statistical analysis

For the reduction in pain intensity (NRS) in the pre-post comparison when using the exergame/conventional training, as well as for the CPGQ, Ffb-H-R, TSK-11 and SF-12, a normal distribution was not present in the data collected and an ordinal scaling was present, thus a Wilcoxon signed-rank test was applied. A Mann–Whitney *U* Test was applied to evaluate the difference between IG and CG of the mean NRS differences in the pre-post comparison per training session. In order to check whether the number of training units was related to pain intensity reduction, a Spearman's rank correlation coefficient was calculated for both groups because of the non-normal distribution. The statistical software SPSS 26 and RStudio were used for the analysis.

## Results

### Study population

**Fig. 3:**
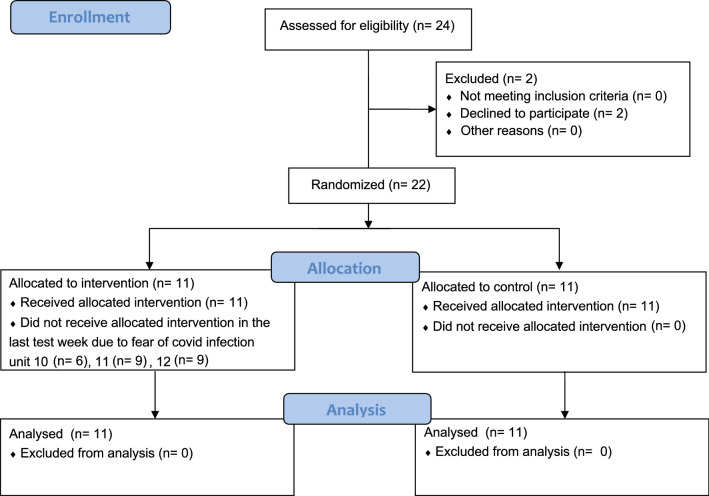
Flow Diagram (Schulz et al. 2010)

The total sample included 22 participants with CBP (Table 1), who met the eligibility criteria and were randomized between January and March 2020. The IG consisted of 11 pain patients aged 67 to 84 years (*M* = 75.0, SD = 5.8), who suffered from back pain for an average of 15.8 years (SD = 12.7). The average pain intensity measured by the NRS was 3.36 (SD = 1.91). The CG included 11 pain patients aged 68 to 84 years (*M* = 75.5, SD = 4.4). The participants had been suffering from back pain for 26.4 years on average (SD = 16.6). The average pain intensity measured by the NRS was 2.91 (SD = 1.64) in the CG. The gender distribution in both groups was balanced (54.5% female). In both groups, the most common diagnosis was lumbar spinal syndrome. There were no serious adverse events reported in either group during the study. The study was terminated as the treatments in the study were completed and the study reached the planned sample size (Fig. 3).

**Table 1:** Study characteristics

Sociodemographic data	Intervention Group	Control Group	*p* value
*Sample size total [n]*	*11*	*11*	
Gender (Female/Male)	8/3	6/5	.659*
Age [M (SD)]	75 (5.80)	75.5 (4.39)	.838^†^
*Highest educational attainment [n]*			.300^‡^
University	6	4	
Advanced technical college certificate	0	2	
High school	3	0	
Secondary school	2	2	
Main school	0	3	
Pain intensity during anamnesis [NRS (SD)]	3.36 (1.91)	2.91 (1.64)	.562^‡^
Duration of back pain [M in years (SD)]	15.8 (18.67)	26.4 (16.57)	.196^†^
*Diagnoses (most common) [n]*			-
Lumbar spine –Syndrome	4	4	
Lumbar disc herniation	1	1	
Thoracic disc herniation	1	0	
cervical disc herniation	1	0	
Facet joint arthrosis	1	1	
Scoliosis	2	2	

*Fisher’s exact test

^†^t-test

^‡^Mann–Whitney-*U*

### Primary outcomes

The summarized results of the applied assessments in the study can be found in Table 2.

**Table 2:** Summary table of results

Variables	Group	Pre intervention Mean (SD)	Pre intervention Median [95% CI]	Post intervention Mean (SD)	Post intervention Median [95% CI]	*p* value	Effect size r
NRS	Intervention	3.55 (2.38)	3.00 [1.95–5.15]	2.91 (2.02)	3.00 [1.55–4.27]	.535	.19
	Control	2.91 (2.38)	3.00 [1.31–4.51]	1.64 (1.50)	1.00 [.63–2.65]	.070	.54
Ffb-H-R	Intervention	73.11 (10.60)	70.83 [65.98–80.23]	81.82 (11.22)	79.16 [74.28–89.36]	.026	.67
	Control	69.80 (16.84)	70.83 [58.49–81.11]	72.73 (15.74)	70.83 [62.15–83.30]	.330	.29
TSK-11	Intervention	19.27 (5.92)	18.00 [15.30–23.25]	17.82 (4.69)	17.00 [14.67–20.97]	.440	.23
	Control	21.55 (6.71)	21.00 [17.04–26.06]	20.73 (8.14)	17.00 [15.26–26.19]	.690	.12
SF-12 physical	Intervention	40.97 (7.83)	42.05 [35.37–46.58]	39.30 (8.01)	40.34 [33.91–44.68]	.575	.18
	Control	35.85 (7.91)	34.07 [30.19–41.51]	37.76 (7.27)	37.63 [32.87–42.65]	.441	.24
SF-12 mental	Intervention	46.44 (10.64)	48.70 [38.83–54.06]	48.39 (7.13)	49.87 [43.60–53.19]	.445	.24
	Control	50.31 (7.66)	53.60 [44.83–55.80]	56.23 (4.77)	56.18 [53.03–59.43]	.011	.81
TUI immersion	Intervention	–	–	19.09	24.00 [13.35–24.83]	–	–

#### Pain intensity progression

**Fig. 4:**
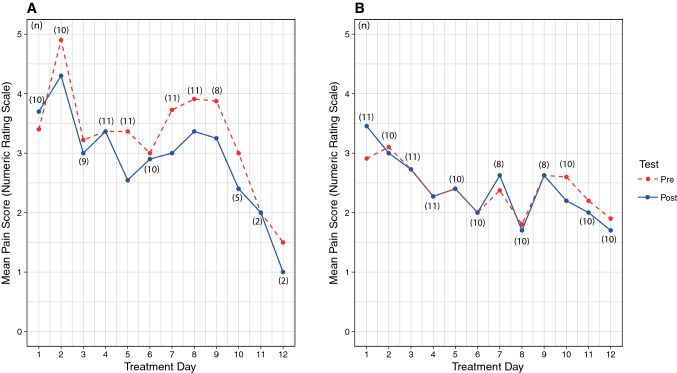
Pain intensity progression during treatment **A** IG, **B** CG

The primary analysis was intention-to-treat and involved all participants who were randomly assigned. The pain intensity decreased over time in both groups. Due to the regulations imposed by the COVID-19 pandemic, some participants in the IG completed the intervention phase early, bringing forward Visit 2 (Fig. 4). Therefore, the progression of pain intensity was compared for both groups between NRS scores measured before the first treatment (Visit 1) and after the last individual treatment performed (Visit 2). The IG completed an average of 9.18 (SD = 1.47) training units and the CG completed an average of 10.81 (SD = 1.6) training units. Participants in the IG rated their pain intensity before the first treatment as $\overline{x }$ = 3.55 (SD = 2.38, 95% CI [1.95, 5.15]) on the NRS. In Visit 2 after the last treatment (Fig. 4), the mean pain intensity in the IG reduced to $\overline{x }$ = 2.91 (SD = 2.02, 95% CI [1.55, 4.27]). Thus, the resulting mean pain intensity reduction in the IG was 0.64 (SD = 3.29, *Z* = −0.62, *p* = 0.535). Participants in the CG assessed their pain intensity before the first treatment (Visit 1) as $\overline{x }$ = 2.91 (SD = 2.38, 95% CI [1.31, 4.51]). In Visit 2 after the last treatment, the mean pain intensity in the CG reduced to $\overline{x }$ = 1.64 (SD = 1.50, 95% CI [0.63, 2.65]). In the CG, a higher pain intensity reduction could be observed than in the IG, with a mean difference of 1.27 (SD = 2.24, *Z* = −1.79, *p* = 0.07).

**Fig. 5:**
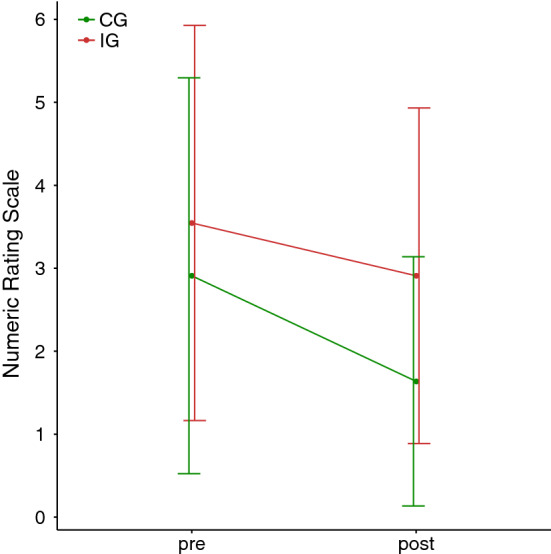
Pre/post comparison of the pain intensity in the intervention and control group with error bars

In order to check whether the number of training units was related to pain intensity reduction, a Spearman's rank correlation coefficient was calculated for both groups. The results showed a weak correlation (IG: *r*_s_ = −0.154, *p* = 0.550, *n* = 11; CG: *r*_s_ = 0.205, *p* = 0.650, *n* = 11), however, both values of the correlations are not significant (Fig. 5).

**Fig. 6:**
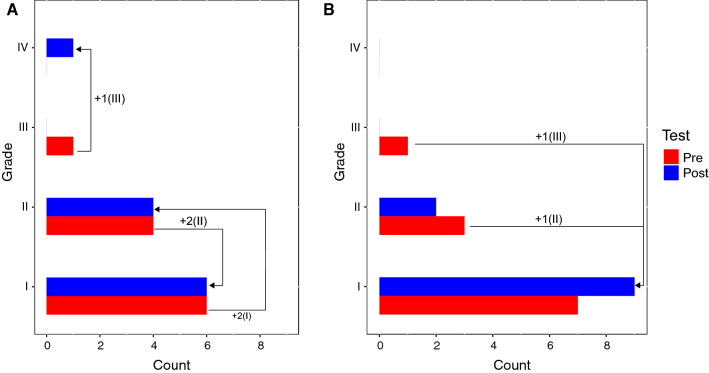
Pre/Post comparison of the global severity of chronic pain in subjects assessed with the CPGQ. **A** IG, **B** CG

The NRS mean difference of participants in the pre-post comparison per training session, measured before the session and immediately after, was calculated from 101 sessions in the IG and 119 sessions in the CG. To evaluate the difference between IG and CG of the mean NRS differences in the pre-post comparison per training session a Mann–Whitney U Test was applied. The test revealed insignificant differences in the mean NRS differences of the IG (Median = −0.57, *n* = 11) and CG (Median = −0.06, *n* = 11), *U* = 36.00, *z* = 1,610, *p* = 0.116, *r* = 0.34. The null hypothesis: the distribution of the mean NRS difference in the pre-post comparison across the two groups is identical, is therefore retained (Fig. 6).

#### Severity of chronic pain

**Fig. 7:**
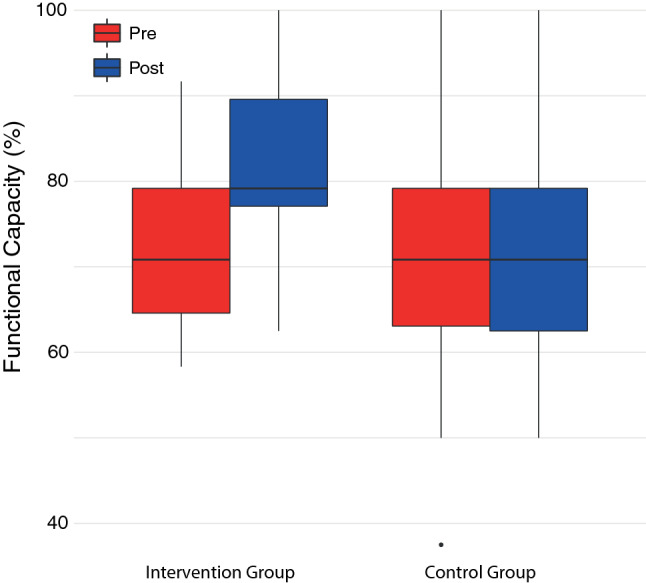
Results of the Ffb-H-R in both groups in pre/post comparison. Values around 70% functional capacity are considered moderate functional impairment, values from 80 to 100% correspond to normal functional capacity, which was achieved in the IG after the treatment

In the IG, six participants could be classified as grade I before the first treatment (Visit 1). After the intervention (Visit 2), no changes could be detected in grade I. Four participants were classified as grade II and thus remained at the same classification after the intervention. Only one participant showed any change, from grade III before the intervention to grade IV after the intervention. In the CG, seven participants could be classified as grade I before the first treatment (Visit 1), and after the four-week conservative therapy (Visit 2), nine participants were subsequently classified as grade I. The number of grade II participants was reduced from three to two. Prior to the intervention (Visit 1), the CG included one participant at grade III and none at grade IV. After the therapy there were no grade III participants (Fig. 7).

#### Functional capacities

The results of the Ffb-H-R showed that a significant improvement in subjective functional capacity in the IG was perceived in the context of basal everyday activities from the mean functional capacity in Visit 1, $\overline{x }$ = 73.11% (SD = 10.60, 95% CI [65.98, 80.22]), to Visit 2, $\overline{x }$ = 81.82% (SD = 11.22, 95% CI [74.28, 89.36]). The mean difference (MD) was 8.71% (*Z* = −2.23, *p* = 0.026), which was significant. In the CG the functional capacity improved from Visit 1, $\overline{x }$ = 69.80% (SD = 16.84, 95% CI [58.49, 81.11]), to Visit 2, $\overline{x }$ = 72.73% (SD = 15.74, 95% CI [62.15, 83.30]), resulting in a MD of 2.93%. However, there was no significant difference in functional capacity in the pre/post comparison of the CG (*Z* = −0.97, *p* = 0.33).

#### Fear-avoidance beliefs

**Fig. 8:**
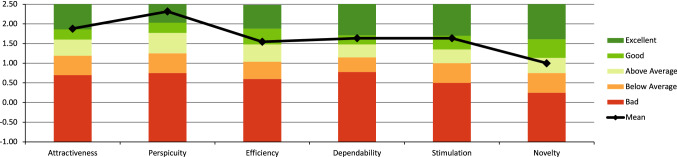
UEQ scales of the evaluated VR system compared to benchmarks

The IG results showed a reduction from 19.27 (SD = 5.92, 95% CI [15.30, 23.25]) to 17.82 (SD = 4.69, 95% CI [14.67, 20.97]) points (MD: 1.45, *Z* = −0.77, *p* = 0.44) on the TSK-11 in the pre/post comparison. The CG showed a reduction in pain-related fear from 21.55 (SD = 6.71, 95% CI [17.04, 26.06]) to 20.73 points (SD = 8.14, 95% CI [15.26, 26.19]) (MD: 0.82, *Z* = −0.40, *p* = 0.69). Neither group showed significant results (Fig. 8).

### Secondary outcomes

#### General physical and mental health

In the IG, before the treatment (Visit 1), the physical cumulative score on the SF-12 was 40.97 (SD = 7.83, 95% CI [35.37, 46.58]) and the mental cumulative score was 46.44 points (SD = 10.64, 95% CI [38.83, 54.06]). After the application of the four-week multimodal VR therapy (Visit 2), a value of 39.30 points (SD = 8.01, 95% CI [33.91, 44.68]) was measured on the physical cumulative scale and a value of 48.39 points (SD = 7.13, 95% CI [43.60, 53.19]) on the mental cumulative scale. Neither change was significant (physical *p* = 0.575; mental *p* = 0.445). In the CG, at Visit 1, a value of 35.85 points (SD = 7.91, 95% CI [30.19, 41.51])) was measured on the physical cumulative scale and a value of 50.31 points (SD = 7.66, 95% CI [44.83, 55.80]) on the mental cumulative scale. After the application of the four-week multimodal therapy (Visit 2), a score of 37.76 points (SD = 7.27, 95% CI [32.87, 42.65]) was recorded on the physical cumulative scale and 56.23 points (SD = 4.77, 95% CI [53.03, 59.43])) on the mental cumulative scale. The physical cumulative scale showed no significance (*p* = 0.441), whereas a significant increase was recorded on the mental cumulative scale (*p* = 0.011). The mental cumulative scale of the CG showed a significant increase on the pre/post comparison of the SF-12 in both groups.

#### Degree of immersion

Since this scale was only collected after the use of VR, an average score of all participants of the IG was calculated, which was 19.09 points (percentile rank 60–77) (min = 3, max = 28), corresponding to a higher degree of immersion. Only 23% of the reference population achieved a higher value in comparison to the VR group investigated here.

#### User experience

The participants rated the VR system as at minimum above average on each individual subscale of the UEQ. The VR system achieved an ‘excellent’ rating for attractiveness and perspicuity. Efficiency, dependability and stimulation were rated as ‘good,’ while the originality of the VR solution was rated as ‘above average.’

The measured data were subsequently set in relation to benchmark data (Table 3). The benchmark data set contains data from 20,190 participants from 452 studies concerning different products (business software, web pages, web shops, social networks).

**Table 3:** User experience questionnaire results

Scale	Evaluated Prototype compared to benchmark Confidence intervals (*p* = 0.05) per scale				
	Mean	Std. Dev	Conf. interval	Comparison to Benchmark	Interpretation
Attractiveness	1.88	0.79	1.42–2.34	Excellent	In the range of the 10% best results
Perspicuity	2.32	0.78	1.86–2.78	Excellent	In the range of the 10% best results
Efficiency	1.55	0.67	1.15–1.94	Good	10% of results better, 75% of results worse
Dependability	1.64	0.85	1.14–2.14	Good	10% of results better, 75% of results worse
Stimulation	1.64	1.07	1.00–2.27	Good	10% of results better, 75% of results worse
Novelty	1.00	1.24	0.27–1.73	Above average	25% of results better, 50% of results worse

## Discussion

### Main findings

The aim of this unblinded randomized controlled pilot study was to evaluate a four-week multimodal pain management in the form of a VR therapy. We conducted the study with older adults with CBP, in which the IG received movement therapy and psychoeducation in a VR exergame, and the CG received movement therapy as chair-based group exercises and psychoeducation in a real-world group setting.

Although the VR therapy (IG) did not reach the pain intensity reduction of the CG (IG: MD = 0.64; CG: MD = 1.64), the results of the present study showed that a pain intensity reduction can be reached with the current VR exergame prototype, albeit not significantly. In the study of Nees et al. (2020), chronic non-specific back pain patients completed a conventional three-week multidisciplinary pain management program. The mean pain intensity decreased from 5.24 ± 2.08 to 3.80 ± 2.02 (MD: 1.44). The mean pain intensity reduction roughly corresponds to that of our CG. On average, a reduction of approximately two points or approximately 30% on the NRS represents a clinically important difference for chronic low back pain (Farrar et al. 2001; Maughan and Lewis 2010). In the IG, a pain intensity reduction of 18.02% was achieved, whereas the CG reached a reduction of 43.64%. In the pilot study of Darnall et al. (2020), a VR cognitive behavioral therapy treatment for chronic pain led to an average pain intensity reduction of 30% (NRS MD = 1.48 SD = 2.08) in a 21-day treatment period. Considering the pain intensity values after 12 days, similar results were achieved as in our study with the IG. The proof-of-concept study of Alemanno et al. (2019) showed greater pain reduction in the use of a VR headset in conjunction with an exercise therapy for CBP. Patients who underwent a six-week neurorehabilitative treatment (12 sessions) using VR showed a significant average pain reduction of 4.5 on the NRS. Possible explanations for a lower reduction in pain intensity in the CG are, on the one hand, the higher accuracy of the exercise execution without VR headset and, on the other hand, group therapy was offered in the control group. The social aspect should not be neglected in the target group, as the exchange can act like a support group. Participants often came a little earlier or stayed a little longer after the training session to communicate with other group members. This illustrates that a certain group dynamic was present. The social component in particular is an important factor in therapy adherence and should not be underestimated for the target group (Picorelli et al. 2014; Mehra et al. 2016).

The results of the present study showed a significant improvement in the IG (*p* = 0.026) in the subjective functional capacity (Ffb-H-R) in the context of activities of daily living (mobility, personal hygiene, getting dressed and undressed). In the IG after the therapy in VR, the values improved from a functional capacity classified as moderate functional impairment (73.11%) to a normal functional capacity (81.82%).

In terms of fear-avoidance behavior (TSK-11), both groups showed a reduction compared to the data before the four-week intervention, however, without significance. The IG showed a reduction from 19.72 to 17.82 points and the CG reduction from 21.55 to 20.73 points. Part of the Fear-Avoidance model (Leeuw et al. 2007) is catastrophizing pain; studies using VR in CBP also observed a significant decrease in pain catastrophizing over time, which may be consistent with the decreasing results of the TSK-11 score in our study. However, the reference data of people with chronic pain (Hapidou et al. 2012) show a TSK-11 score of 30.4 (SD = 6.6), which is considerably above the achieved pre and post values in the present study. The comparable study data suggest that fear avoidance was underrepresented in our two cohorts.

In both groups, an increase in the mental summative scales was observed in the results of health-related quality of life (SF-12). Only the CG increased its value in the physical cumulative scale of the SF-12. Further, users of the VR system perceived an immersion that can be interpreted as ‘higher degree of immersion’ according to the TUI. The participants of the IG rated the VR system as at least above average in the UEQ. The VR system achieved a ‘excellent’ rating for attractiveness and perspicuity.

An important factor for the treatment outcome of CBP is the adherence to home exercises or exercise behavior outside of the physiotherapy sessions (Mannion et al. 2009). Studies in physiotherapy indicate that higher exercise adherence is associated with improved physical function (Thomas et al. 2002; van Gool et al. 2005). However, maintaining the exercise regimen at home can be difficult for many patients. Slujis et al. (1993), showed that only 35% of participants (*N* = 1,178) performed exercises at home. To increase motivation and adherence, a VR exergame under physiotherapeutic supervision for the treatment of older patients with CBP would be a conceivable adjunctive therapy to multimodal pain management. However, VR therapy should not replace conventional multimodal pain therapy, as its effectiveness is too low according to our study.

### Feasibility

The pilot study demonstrated it would be feasible to conduct a larger RCT study using a multimodal pain management in VR. Nevertheless, some adjustments are required in order to conduct a larger study. One challenge that some participants had when using the VR game was their own body size. For some participants, for example, the arm span was not sufficient to place the vegetables from the conveyor belt into the boxes to the right and left of them during the “vegetable sorting.” A possible solution would be to adapt the exercise by improving the camera and object height integrated into the system. Furthermore, participants shared that the achieved score of some exercises were not immediately or not at all apparent or judged the interpretation of the score as intransparent. In a subsequent VR games, attention should be paid to the visualization of the player's achieved score as well as that of the maximum score to be achieved. Furthermore, some of the test persons noticed that a wrong execution of some exercises did not result in any (negative) consequence (e.g., torn hurdle during the exercise “hurdles”). As a solution, more attention should be paid in future to the game mechanics. Furthermore, it became apparent during the execution of the exercise that the positioning to the respective object shown in VR varied in part and that equal distances to the center of the object were not given (e.g., during the exercise “ball bucket”). This led to the fact that the object could not be grasped precisely. Likewise, in future, the exercise needs to be adapted by adjusting the position of the respective object.

With regard to the voice dialog system, the assignment of voice commands did not function correctly. Voice commands, such as “One more time.” or “Repeat exercise.” did not result in correct execution of the VR software. In addition, some participants criticized the latency between voice recording and execution by the VR software. Last, participants shared that they would like to see more variation in the communication of praise.

Regarding a future RCT, blinding of the assessors, e.g., by blinding the outcome assessment to limit observer bias, also called "detection bias" would be advisable. This was not applied in the pilot study. Further, future studies should examine CBP’s motivation and adherence during the VR training period. Although user experienced was measured in the IG in this study, a comparison to a control group is necessary to compare the results. The user experienced was only considered in the intervention group to explore initial results. For this reason, this would be an interesting outcome for a future RCT.

### Limitations

The interpretation of the study findings is limited because of the small sample size. The study of a demonstrator created in the grant guideline is more of a proof-of-concept than a large-scale clinical trial. The VR system represents a pre-economic demonstrator. For this reason, further investigation with older people with chronic low back pain using multimodal VR therapy is needed. The tested demonstrator needs further adaptation for future successful use and further research with larger samples. Another limitation was the unequal gender distribution in the two samples, although it did not differ significantly. However, considering the prevalence of chronic back pain, 28.0% of women and 17.4% of men aged 70 or older have this condition in Germany (Von Der Lippe et al. 2021). Therefore, a targeted representation of these numbers could be considered in a larger study for both groups. The study duration of four weeks is a minimum for such a pain management program; the system could be tested in further studies over a longer period of time. A bias could have occurred from the fact that the CG received group therapy, which could have led to increased motivation through group dynamic processes. The VR exergame of IG, represented individual therapy, therefore, in future, a more adequate control group would be with individual therapies instead of group therapy or the application of group therapy in the exergame. Due to the onset of the COVID-19 pandemic toward the end of the study, several participants in the IG did not attend the final appointments of the intervention phase through fear of infection. Furthermore, the usability of the system was limited by the handling of the dialog system, which required a learning phase by the participants in the IG. Other technical problems in the IG included connection problems with the base stations and Internet problems, which led to a brief interruption of the image transmission in the VR headset.

## Conclusion

This exploratory pilot study examined the preliminary effectiveness of a VR HMD multimodal therapy in a laboratory setting for older CBP patients. Our findings showed only a significant improvement in the subjective functional capacity (Ffb-H-R) after the completion of a four-week multimodal pain therapy in VR (movement therapy and psychoeducation). In the changes of fear-avoidance beliefs and general physical and mental health, no significance was found in either group. Although the VR therapy did not reach the pain intensity reduction of the conventional multimodal pain therapy, the results of the present study showed that a pain intensity reduction can be achieved with the current VR therapy. The users perceived a higher degree of immersion and rated the user experience as mostly good and excellent in attractiveness and perspicuity.

The pilot study has provided important insights for further studies. Further research is needed to assess the motivation and adherence. In its current state, the prototype would only be a viable option for the treatment of elderly patients with CBP under physiotherapeutic supervision. In general, such a solution would be considered as an adjunctive therapy to the multimodal pain management, but cannot be used as a replacement.

## Data Availability

The datasets generated are available from the corresponding author on reasonable request.
